# Longitudinal associations of depression, anxiety, and stress among healthcare workers assisting patients with end-stage cancer during the COVID-19 pandemic: the moderator role of emotional exhaustion

**DOI:** 10.1186/s40359-024-01851-1

**Published:** 2024-06-20

**Authors:** Alberto Sardella, Alessandro Musetti, Christian Franceschini, Maria C. Quattropani, Vittorio Lenzo

**Affiliations:** 1 Department of Educational Sciences, University of Catania, Catania, Italy; 2 Department of Humanities, Social Sciences and Cultural Industries, University of Parma, Parma, Italy; 3 Department of Medicine and Surgery, University of Parma, Parma, Italy

**Keywords:** Clinical psychology, COVID-19, Longitudinal, Burnout, Depression, Anxiety, Stress

## Abstract

**Background:**

This study aimed to analyze the moderating role of emotional exhaustion in the relationships between longitudinal associations of depression, anxiety, and stress among healthcare workers assisting end-of-life cancer patients during the COVID-19 pandemic.

**Methods:**

A longitudinal study involving a final sample of 122 healthcare workers (61.5% females, mean age = 39.09 ± 11.04 years) was conducted. These participants completed the Maslach Burnout Inventory (MBI) and the Depression Anxiety Stress Scales-21 (DASS-21). Results: Results of correlation analysis showed that emotional exhaustion was correlated with both T1 and T2 measures of depression, anxiety, and stress. Results of the moderation analysis indicated that emotional exhaustion moderated the relationships between consecutive measures of depression and anxiety. Each of the moderation models explained about half of the variance for depression and anxiety. When considering stress, results did not show a moderating role for emotional exhaustion.

**Conclusions:**

Overall, the results of this study highlight that emotional exhaustion moderated depression and anxiety over time. Psychological interventions to promote psychological mental health among healthcare workers assisting patients with end-stage cancer should carefully consider these findings.

## Background

In Italy, recent estimates reported more than 180,000 cancer deaths [1] and almost 40,000 received palliative home care [2]. When assisting their patients, healthcare workers face a prolonged stressor that may lead them to develop burnout, as well as depression, anxiety, and stress [3, 4]. It is worth noting that they faced further prolonged and unknown stress factors in recent years, such as the COVID-19 outbreak. By exposing them to stressful conditions (for instance, a high risk of infection), the psychological impact of the pandemic has increased the risk of burnout among healthcare workers and compromised their perception of safety [5–7]. A 1-year observational study, indeed, found that burnout and anxiety remained stable one year after the pandemic onset among Canadian healthcare workers, while symptoms of depression and posttraumatic stress disorder diminished [8]. Another study, focusing on the psychological response of Italian nurses during the first and the second wave of the pandemic, found persistent emotional distress, even though personality traits seem to mediate the long-term experienced stress [9]. From the perspective of avoiding the onset of burnout syndrome, prevention is paramount. Since Freudenberger [10] described physical and behavioral signs of burnout among professional volunteers and paid staff members, a flourishing literature has attempted to clarify the underlying process responsible for the onset of the syndrome [11–13]. Generally, burnout constitutes a psychological syndrome consequent to prolonged work-related stress [14]. It embeds three key factors, namely emotional exhaustion, depersonalization, and reduced personal accomplishment. Emotional exhaustion is usually deemed as an individual response to stress, together with symptoms of fatigue, depletion, and weakness. Depersonalization is characterized by loss of compassion, cynicism, and more generally by a negative relational attitude towards others and colleagues. Lastly, feelings of inadequacy at work, along with loss of self-confidence, feature personal accomplishment. Burnout develops gradually over time, though it may remain hidden [15], while these three factors were frequently outlined in sequential stages originating from high stress at work. Of particular significance is that the emotional exhaustion factor has been considered the core manifestation of burnout [16, 17]. Indeed, Maslach and Leiter [18] have argued that exhaustion is robustly correlated with stress symptoms and, consequently, it is a stronger predictor of stress-related outcomes than depersonalization and personal accomplishment.

At this point, a few further considerations should be made on burnout among healthcare workers involved in critical care and end-of-life care. Burnout in healthcare workers providing palliative represents a significant concern, even though its prevalence varies widely [19] Nonetheless, caring for patients at the end-of-life stage is acknowledged as a weighty experience [20, 21], as it entails the involvement of a series of psychological, emotional, and even moral factors [22]. The frequent exposure to death, and even before the exposure to the prolonged suffering of terminal patients, and the burden to share the loss and mourning of a loved one with families, have been indicated among the factors that most contribute to high psychological distress in healthcare workers in such contexts of treatment [23, 24], with inevitable negative consequences also for the quality of care [25]. Consistently, discussing burnout among health workers involved in the care of end-of-life patients acquires a deeper relevance, since these carers appear more vulnerable, or at least vulnerable in a different way, compared to other professional figures not directly involved in end-of-life care [26, 27].

Another caveat concerns the relevance of differential diagnosis between burnout and depression, already highlighted by Freudenberger [10]. The growing recent literature on the epistemic status of burnout has pointed out that the distinction with depression remains theoretically unclear [28]. While, on one hand, there is still a lack of consensus on the diagnosis of burnout syndrome, on the other hand, the heterogeneous clinical manifestations of depressive disorders may overlap with it. Besides supporting a better understanding of the interplay between burnout and depression, knowledge of the other symptoms frequently occurring would have many other implications for prevention. Since burnout originates from prolonged stress at work and many times it can trigger anxious feelings, Koutsimani and colleagues [29] have investigated the relationship of burnout with depression and anxiety in a recent systematic review and meta-analysis. Findings of this study converged to suggest that these constructs are distinct phenomena, though they may share some characteristics. The above-mentioned authors also indicated the need for a longitudinal design to deepen the existing relationships among burnout, depression, and anxiety. In our opinion, this denotes a crucial goal for future studies from both theoretical and practical points of view. On one hand, the predictive role of burnout for the worsening of psychological symptoms over time would be clarified, also supporting a theoretical model of psychological trajectories that can be extended to different health professions. On the other hand, longitudinal evidence can be useful in order to implement tailored and person-centered psychological interventions, as well as to promote a positive work experience, by improving primary and secondary prevention through a more accurate disclosure of psychological problems. At the very least, in pursuing this question, the relationship with stress should be also considered on the ground that burnout stems from chronic emotional and interpersonal stressors in the work environment [16].

**Fig. 1:**
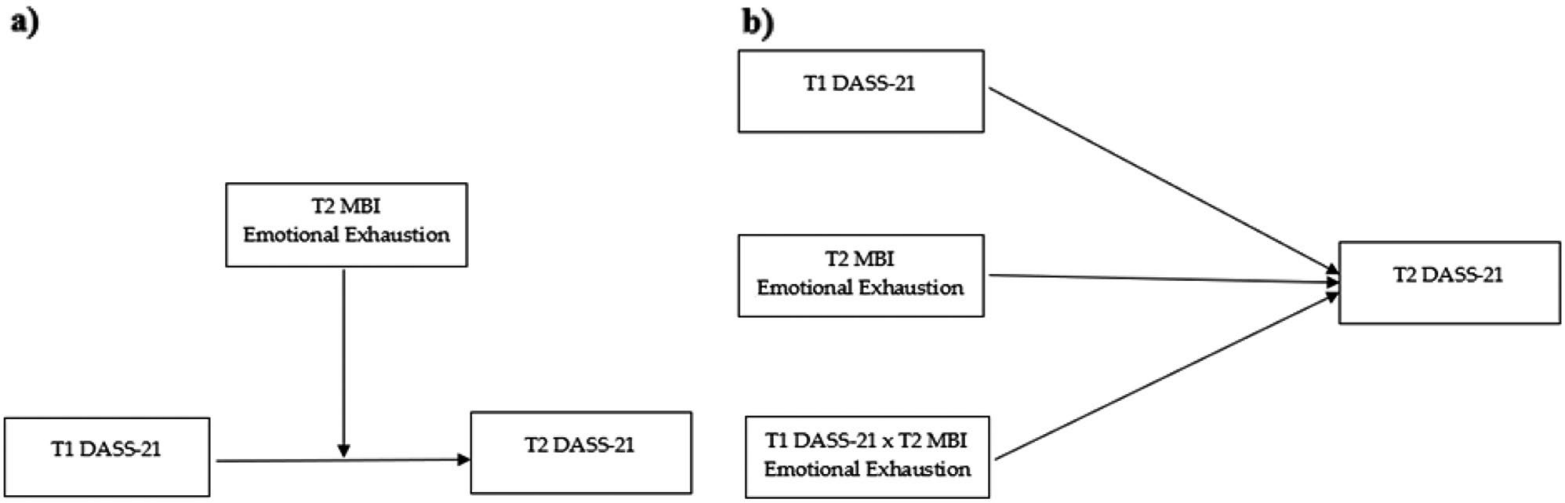
Conceptual **(a)** and statistical **(b)** diagram of the moderated role of Emotional Exhaustion in the relationship between T1 and T2 DASS-21 Scales (i.e., Depression, Anxiety, and Stress)

Based on these premises, this study has two main aims as follows:

1. To examine the relationships between burnout, depression, anxiety, and stress, as well as differences between the first and the second wave. Grounded on the literature, we hypothesize significant positive correlations among the observed variables and stable depression, anxiety, and stress levels during the second wave.
2. To examine the moderating role of emotional exhaustion in the relationship between two longitudinal measurements of depression, anxiety, and stress in healthcare workers. The conceptual model depicting the observed variables in a moderate relationship is illustrated in Fig. 1. We hypothesized that emotional exhaustion is a moderator of the relationship between longitudinal associations of depression, anxiety, and stress. Emotional exhaustion is a crucial core factor of burnout, and it frequently configures and characterizes the work experience of healthcare professionals involved in end-of-life care. Such work experience is also exacerbated by increased psychological distress (i.e., anxiety, depression, and stress symptomatology). Therefore, in line with our research hypothesis, among this specific vulnerable group of healthcare professionals, the trajectory of psychological distress over time can be moderated by the perceived emotional exhaustion levels.

## Methods

### Participants and procedure

This study was approved by the Research Ethics Committee for Psychological Research of the University of Messina (n. 38,518). The study was conducted in accordance with the 1964 Declaration of Helsinki and its later amendments. The privacy of the participants was guaranteed in accordance with the European Union General Data Protection Regulation 2016/679. Participants were enrolled via an online announcement supported by the Local Health Unit of the Italian National Health Service (INHS) and addressed to healthcare workers assisting end-of-life cancer patients. The announcement linked subjects who were interested in an external page with information and consent to participate in the study. More specifically, participants were asked to provide an email contact and to create an identification code to anonymize it. The informed consent form displayed two options (“Yes” or “No”). Participants who chose “Yes” accessed the survey page. After indicating consent, the URL of the Google Form was accessible, and each participant needed to insert the identification code earlier created. The identification code was also required to access and complete the form for T2 assessment. All participants gave informed consent before participating and completed the survey anonymously. Besides the self-report instruments, the online survey encompasses a series of questions to collect information such as age, gender, relationship status, profession, years of professional experience, and contact with COVID-19 patients. The questionnaire was set to continue only when each option was concluded before the final submission, even though each subject could leave the survey at any time. The T1 data was gathered during the first Italian lockdown and, specifically, from March to May 2020. The T2 data was gathered during the second wave of the pandemic and, specifically, from October 2020 to February 2021. DASS-21 was administered in both T1 and T2 assessments, while MBI was only in the latter. A total of 218 subjects consented to participate in the T1 measurement, whereas 122 completed the T2 measurement.

### Measures

The Maslach Burnout Inventory (MBI) [30] measures symptoms of burnout. The MBI consists of 22 items on a 7-point scale ranging from 0 (“never”) to 6 (“every day”). Items are grouped into three subscales: Emotional Exhaustion (EE), assessing reduced energy, emotional and cognitive withdrawal from the job; Depersonalization (D), measuring the lack of engagement, cynicism, and withdrawal from the patients; Personal Accomplishment (PA), measuring the capacity to face problems at work and perception of having a positive influence on others. In the current study, the Italian version of MBI was used [31]. Only the EE subscale has been used for moderation analyses for this study. In this study, the reliability coefficients of Cronbach’s alpha were 0.77 for EE, 0.53 for DP, and 0.51 for PA.

The Depression Anxiety Stress Scale – 21 (DASS-21) [32] measures depression, anxiety, and stress. The DASS-21 consists of 21 items on a 4-point Likert scale ranging from 0 (“never”) to 3 (“always”). Items are clustered into three subscales: DASS-21 depression, DASS-21 anxiety and DASS-21 stress. DASS-21 depression measures symptoms of depression such as dysphoria, anhedonia, low self-esteem, lack of interest, and passivity (e.g., “I felt down-hearted and blue”). DASS-21 anxiety measures somatic and subjective symptoms of anxiety (e.g., “I was aware of dryness of my mouth”); DASS-21 stress measures prolonged arousal, psychomotor agitation, irritability, and psychological tension (e.g., “I felt that I was using a lot of nervous energy”). In this study, the Italian version of DASS-21 showing excellent psychometric properties was used [33]. Reliability coefficients of Cronbach’s alpha for the present sample were 0.81 for T1 DASS-21 depression, 0.86 for T2 DASS-21 depression, and 0.83 for T1 DASS-21 anxiety, 0.86 for T2 DASS-21 anxiety, 0.85 for T1 DASS-21 stress, and 0.92 for T2 DASS-21 stress.

### Statistical analysis

The data were analyzed using SPSS v. 26 (IBM, Armonk, NY, USA) statistical software and the Process Macro for SPSS [34]. Descriptive results were outlined as frequencies (*%*), mean scores, and standard deviations. An independent *t*-test was used to compare the DASS-21 depression, DASS-21 anxiety, and DASS-21 stress scores in T1 and T2. Relationships between DASS-21 and MBI were performed with Pearson product-moment correlation coefficients. Subsequently, three distinct moderation analyses were performed to examine the role of Emotional exhaustion in moderating the effect of T1 depression, anxiety, and stress on T2 measurements. Age and work experience were added as covariates. In each moderation analysis, the effect of interaction was decomposed through simple slope analysis at low (-1 *SD*), medium, and high (+ 1 *SD*) values of the moderator.

## Results

### Demographic and work-related characteristics of the sample

Table 1 shows the demographic characteristics of the final sample. The sample consisted of 75 females and 47 males, ranging in age from 23 to 72 years (*M* = 39.09 ± 11.04). Most of them were married or in a steady relationship (75.4%, *n* = 92). With regard to profession, 27.9% (*n* = 34) of the participants were nurses, 21.3% were healthcare assistants (*n* = 26), 13.1% were physicians (*n* = 16), 12.3% were physiotherapists (*n* = 15), 9% were clinical psychologists (*n* = 11), 5.7% were speech therapists (*n* = 7), 4.1% were social workers (*n* = 5), and 6.5 other health professions (*n* = 8). In both the measurements, they declared to care for end-of-life cancer patients. With regard to the T2 measurement, only 7.4% (*n* = 9) of the healthcare workers assisted end-of-life cancer patients with COVID-19 during the last two weeks, while 13.9% (*n* = 17) had an infected acquaintance or loved one, and 10.7% (*n* = 13) had been in quarantine.

**Table 1:** Demographic and work-related characteristics of the sample

Variable	M	SD	*n*	Percentage
Age (in years)	39.09	11.04		
Gender Male Female			47 75	38.5% 61.5%
Marital status Married or in a steady relationship Single, widowed, or divorced			92 30	75.4% 24.6%
Work experience in years	10.81	9.56		
Assisted COVID-19 patients during the last two weeks Yes No			9 112	7.4% 91.8%
Infected acquaintances or loved ones during the last two weeks Yes No			17 105	13.9% 86.1%
Quarantine for COVID-19 during the last two weeks Yes No			13 108	10.7% 88.5%

### Depression, anxiety, and stress differences between T1 and T2

There were no significant differences between T1 and T2 for DASS-21 depression (T1: *M* = 2.16, *SD* = 2.47 and T2: *M* = 2.08, *SD* = 2.86, *t*(121) = 0.37; *p* = 0.71), DASS-21 anxiety (T1: *M* = 1.46, *SD* = 2.24 and T2: *M* = 1.30, *SD* = 2.37, *t*(121) = 0.69; *p* = 0.49), and DASS-21 stress (T1: *M* = 4.19, *SD* = 3.12 and T2: *M* = 4.01, *SD* = 3.83, *t*(121) = 0.55; *p* = 0.58).

### Descriptive and correlational analyses between burnout, depression, anxiety, and stress

Table 2 shows the descriptive statistics and results of correlation analyses. The T1 and T2 measurements of the DASS-21 subscales were all correlated with each other. Specifically, T1 DASS-21 depression was positively correlated with its T2 measurement (*r* = 0.59; *p* < 0.01), T2 DASS-21 anxiety (*r* = 0.35; *p* < 0.01), and T2 DASS-21 stress (*r* = 0.36; *p* < 0.01). T1 DASS anxiety was positively correlated with its T2 measurement (*r* = 0.41; *p* < 0.01), T2 DASS-21 depression (*r* = 0.38; *p* < 0.01), and T2 DASS-21 stress (*r* = 0.28; *p* < 0.01). T1 DASS-21 stress was positively correlated with its T2 measurement (*r* = 0.47; *p* < 0.01), T2 DASS-21 depression (*r* = 0.46; *p* < 0.01), and T2 DASS-21 anxiety (*r* = 0.40; *p* < 0.01). Additionally, MBI EE was positively correlated with T1 DASS-21 depression (*r* = 0.39; *p* < 0.01), anxiety (*r* = 0.24; *p* < 0.01), and stress (*r* = 0.43; *p* < 0.01). T2 MBI EE was also positively correlated with T2 DASS-21 depression (*r* = 0.64; *p* < 0.01), anxiety (*r* = 0.54; *p* < 0.01), and stress (*r* = 0.70; *p* < 0.01). Lastly, T2 MBI D was positively correlated with T2 DASS-21 depression (*r* = 0.28; *p* < 0.01), anxiety (*r* = 0.21; *p* < 0.01), and stress (*r* = 0.31; *p* < 0.01).

**Table 2:** Descriptive and Correlational Analyses between burnout, depression, anxiety, and stress

Variable	Min	Max	M	SD	1	2	3	4	5	6	7	8
1. T1 DASS-21 depression	0	12	2.16	2.47								
2. T1 DASS-21 anxiety	0	13	1.46	2.24	0.64**							
3. T1 DASS-21 stress	0	14	4.19	3.12	0.68**	0.67**						
4. T2 DASS-21 depression	0	14	2.08	2.86	0.59**	0.38**	0.46**					
5. T2 DASS-21 anxiety	0	15	1.3	2.37	0.35**	0.41**	0.40**	0.77**				
6. T2 DASS-21 stress	0	19	4.01	3.83	0.36**	0.28**	0.47**	0.77**	0.75**			
7. T2 MBI EE	0	51	9.74	10.26	0.39**	0.24**	0.43**	0.64**	0.54**	0.70**		
8. T2 MBI D	0	14	2.81	3.7	0.17	0.09	0.17	0.28**	0.21*	0.31**	0.37**	
9. T2 MBI PA	0	48	40.45	9.93	-0.09	-0.11	-0.03	0.06	0.09	0.12	0.07	0.01

Notes T2 MBI EE = T2 MBI Emotional Exhaustion; T2 MBI D = T2 MBI Depersonalization; T2 MBI PA = T2 MBI Personal Accomplishment;^*^p<.05;^**^p<.01

### Moderation of T1 and T2 depression, anxiety, and stress by emotional exhaustionDepression

Three separate moderation models were carried out to test whether Emotional Exhaustion moderated the effect of the T1 measurements of depression, anxiety, and stress on the T2 assessments. Table 3 shows the results of moderation analysis for T1 and T2 depression measurements by emotional exhaustion. Results indicated that both T1 DASS-21 depression (*B* = 0.43; *p* < 0.01) and T2 MBI Emotional Exhaustion (*B* = 0.12; *p* < 0.01) had a significant effect on T2 DASS-21 depression. Moreover, the interaction between T1 DASS-21 depression and T2 MBI Emotional Exhaustion (*B* = 0.01; *p* < 0.01) was significant. Indeed, as shown in Table 3, T2 MBI Emotional Exhaustion fully moderated the relationship between T1 and T2 DASS-21 depression scores. No statistical significance was found for covariates.

**Fig. 2:**
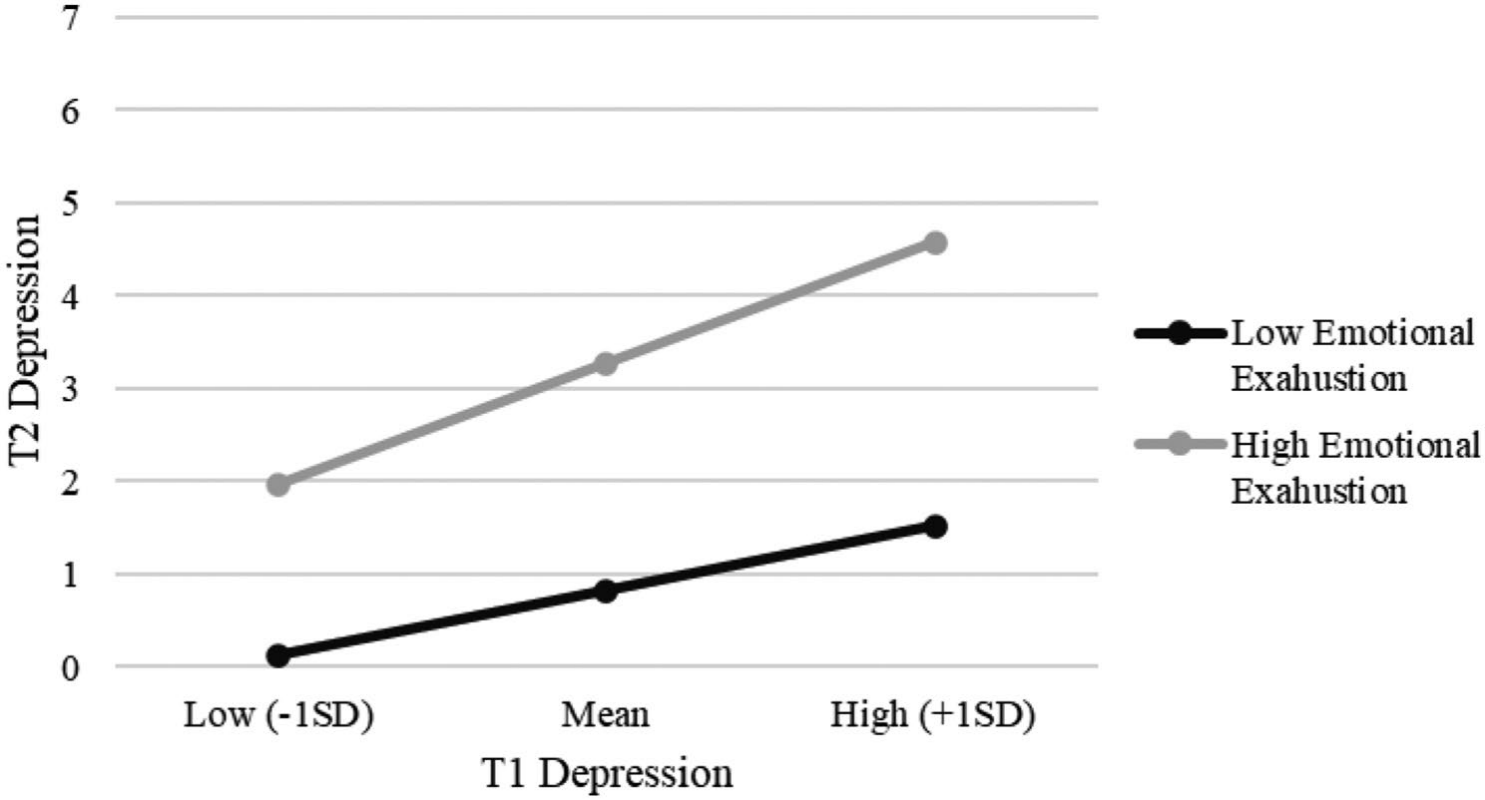
Interaction between T1 Depression and Emotional Exhaustion for T2 Depression among healthcare workers

Figure 2 represents the different slopes for the conditional effect of T1 DASS-21 depression on its T2 measurement highlighting the role of the T2 MBI Emotional Exhaustion factor. The final model explained more than half of the variance for the T2 DASS-21 depression score.

**Table 3:** Emotional exhaustion moderates the relationship between T1 and T2 Depression measurements

	B	SE	T	95% CI
Intercept	1.95	0.18	10.60**	[1.59, 2.32]
T1 DASS-21 depression	0.43	0.08	5.46**	[0.27, 0.58]
T2 MBI EE	0.12	0.02	6.14**	[0.08, 0.16]
T1 DASS-21 depression x T2 MBI EE	0.01	0.01	2.13**	[0.01, 0.03]
Regression Model R^2^ = 0.56**				
Δ R^2^ = 0.02*				
Conditional effects of T1 DASS-21 Depression				
Low T2 MBI EE (-1SD = -9.74)	0.30	0.11	2.77**	[0.09, 0.51]
Medium T2 MBI EE (M = 0)	0.43	0.08	5.46**	[0.27, 0.58]
High T2 MBI EE (+ 1SD = 10.26)	0.56	0.09	6.24**	[0.39, 0.74]

Notes: T2 MBI EE = T2 MBI Emotional Exhaustion. **p < 0.01

### Anxiety

Table 4 displays the results of moderation analysis for T1 and T2 anxiety measurements by emotional exhaustion. Results showed that both T1 DASS-21 anxiety (*B* = 0.28; *p* < 0.01) and T2 MBI Emotional Exhaustion (*B* = 0.09; *p* < 0.01) had a significant effect on T2 DASS-21 anxiety. The interaction between T1 DASS-21 anxiety and T2 MBI Emotional Exhaustion (*B* = 0.03; *p* < 0.01) was also statistically significant. Specifically, medium and high levels of T2 MBI Emotional Exhaustion moderated the relationship between T1 and T2 DASS-21 anxiety scores. No statistical significance was found for covariates.

**Fig. 3:**
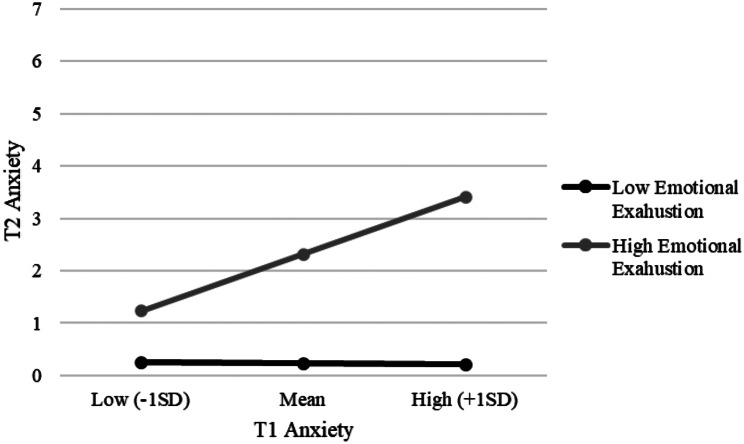
Interaction between T1 Anxiety and Emotional Exhaustion for T2 Anxiety among healthcare workers

Figure 3 displays the different slopes for the conditional effect of T1 DASS-21 anxiety on its T2 measurement highlighting the role of the T2 MBI Emotional Exhaustion factor. The final model explained slightly less than half of the variance for the T2 DASS-21 anxiety score.

**Table 4:** Emotional exhaustion moderates the relationship between T1 and T2 anxiety measurements

	B	SE	T	95% CI
Intercept	1.14	0.17	6.86**	[0.81, 1.46]
T1 DASS-21 anxiety	0.28	0.07	3.77**	[0.13, 0.43]
T2 MBI EE	0.09	0.02	5.77**	[0.06, 0.13]
T1 DASS-21 anxiety x T2 MBI EE	0.03	0.01	4.12**	[0.02, 0.04]
Regression Model R^2^ = 0.45**				
Δ R^2^ = 0.08				
Conditional effects of T1 DASS-21 Anxiety				
Low T2 MBI EE (-1SD = 9.74)	-0.01	0.11	-0.1	[-0.23, 0.20]
Medium T2 MBI EE (M = 0)	0.28	0.07	3.77**	[0.13, 0.43]
High T2 MBI EE (+1SD = 10.26)	0.59	0.1	5.91**	[0.39, 0.79]

Notes: T2 MBI EE = T2 MBI Emotional Exhaustion. **p < 0.01

### Stress

Table 5 illustrates the results of moderation analysis for T1 and T2 stress measurements by emotional exhaustion. Results revealed that both T1 DASS-21 stress (*B* = 0.26; *p* < 0.01) and T2 MBI Emotional Exhaustion (*B* = 0.24; *p* < 0.01) had a significant effect on T2 DASS-21 stress. However, the interaction between T1 DASS-21 Stress and T2 MBI Emotional Exhaustion was not statistically significant. Lastly, no statistical significance was found for covariates.

**Table 5:** Emotional exhaustion moderates the relationship between T1 and T2 Stress measurements

	B	SE	t	95% CI
Intercept	4.08	0.26	15.63**	[3.56, 4.59]
T1 DASS-21 Stress	0.26	0.09	3.01**	[0.09, 0.43]
T2 MBI EE	0.24	0.03	8.26**	[0.18, 0.29]
T1 DASS-21 Stress x T2 MBI EE	0.01	0.01	-0.69	[-0.02,0.01]
Regression Model R^2^ = 0.53**				

Notes: T2 MBI EE = T2 MBI Emotional Exhaustion. Conditional effects of T1 DASS-21 stress are not present because the interaction (T1 DASS-21 stress x T2 MBI EE) was not significant, CI = confidence interval. **p < 0.01

## Discussion

Taking care of patients with chronic and/or terminal medical conditions is a difficult experience, marked by a high psychological burden for careers [13, 35, 36]. Additionally, when compared to the other professional categories, healthcare workers showed a higher risk of burnout and other stress-related psychological symptoms [7]. Therefore, understanding the role of emotional exhaustion in the risk of psychopathology among healthcare workers assisting patients with end-stage cancer during the pandemic would have fundamental importance for prevention. Of particular interest is that our findings are based on a longitudinal approach, while most of the research is cross-sectional [29]. In this vein, this study aimed to explore the interplay between longitudinal associations of depression, anxiety, stress, and emotional exhaustion among healthcare workers assisting end-of-life cancer patients. This general purpose was addressed through two specific aims.

Primarily, we sought to examine the associations between burnout, depression, anxiety, and stress. Perhaps not surprisingly, we found that the longitudinal associations of emotional distress factors were significantly correlated with each other. It is, after all, a further confirmation of what Lovibond [37] has demonstrated in his previous work. Indeed, it turned out that depression, anxiety, and stress did not vary consistently from 3 to 8 years after the first evaluation among a sample composed of healthy subjects. Additionally, findings of Lovibond’s study showed that for each T2 assessment, the highest correlation coefficient was with its correspondent T1 depression, anxiety, and stress evaluation [37]. Relatedly, our findings showed comparable associations in our sample consisting of healthcare workers. The stable reported symptoms of psychopathology across the measurements seem coherent with previous findings. Authors of these studies argued that the restrictive measures characterizing lockdown as well as the uncertainty of its duration could be the causes [38–40].

Nonetheless, we also investigated the relationships of depression, anxiety, and stress with burnout syndrome. Admittedly, most of the researchers have attempted to clarify the interplay between depression and burnout. While the former is related to a specific and persistent work-related stress [14], the latter represents a psychiatric syndrome that may onset without the occurrence of a prolonged stressor [41]. Together with significant overlap, the stigma associated with depression may contribute to make burnout a more acceptable diagnosis for healthcare workers [42].

Some authors have argued that emotional exhaustion represents the core element of burnout [43]. In their review, additionally, Bianchi and colleagues [28] concluded that emotional exhaustion showed the highest correlation coefficients of the other burnout factors (i.e., depersonalization and reduced personal accomplishment). Showing the presence of a significant correlation coefficient between exhaustion and longitudinal measurement of depression, the findings of the current study are consistent with this framework. Postulating a close relationship between depression and burnout, however, did not allow us to establish if they are distinguishable or simply overlap. At the very least, the lack of consensus criteria for burnout syndrome worsens this work of understanding. Forthcoming research adopting a longitudinal approach is also paramount. To date, even though some common symptom such as loss of energy suggests a certain degree of overlap, meta-analytic evidence indicates that burnout and depression are different constructs [29]. At a glance, it may be useful to consider anxiety and depression in a more complex view of this much-debated topic. Anxiety represents one of the most common psychological symptoms among the general population. Therefore, that many healthcare workers may be at risk of developing symptoms of anxiety is not surprising in light of the ongoing COVID-19 pandemic [3]. Most research has shown that anxiety and burnout levels were high and stable over the time of the pandemic [8, 9], especially in frontline healthcare workers during the first wave [44].

Our findings also revealed significant relationships between emotional exhaustion and both T1 and T2 anxiety evaluations, even though the latter showed a higher correlation coefficient than the former. A similar finding has been also found by Zhou and colleagues [45] in a sample of 1274 physicians. When considering this relationship, another study focusing on death anxiety among hospice social workers showed similar results [46]. That is to say, although the COVID-19 pandemic has contributed to increasing their levels among healthcare workers, the relationship between anxiety and burnout should be considered as a proven fact. The interpretation of these findings appears complex insofar as several explanations are equally plausible. As argued by Maslach and colleagues [16], on one hand, healthcare workers who have pre-existing mental problems may be more at risk of developing burnout. On the other hand, mental problems may be the consequence of psychological burnout, especially in times of pandemic. Because of its duration of more than 1 year, indeed, the COVID-19 pandemic can be considered a long-term stress with a profound impact on the population [47]. Once again, the use of longitudinal design constitutes a way to better understand the causality between these aspects. Deeply understanding these relationships also needs to consider stress among healthcare workers with more concern. Despite the high heterogeneity in burnout literature, since Freudenberger’s seminal work [10] researchers converged in highlighting that chronic stress at work portrays the initial stage. As revealed by our results, both T1 and T2 stress measurements were significantly correlated with emotional exhaustion. Not unexpectedly then, many studies have found similar results. For example, a study involving a sample of 426 primary care physicians found a significant correlation between burnout and job stress [48]. Relatedly also, another study focusing on a sample of 527 nurses from 41 hospitals in China, found that high levels of burnout were strictly related to work-related stress [49]. However, it is worth highlighting that longitudinal design may allow us to hone our understanding of these findings insofar as very few studies have addressed this topic [29].

In this vein, the second aim of this study was to verify if emotional exhaustion impinges on the longitudinal relationships of depression, anxiety, and stress. To the best of our knowledge, there is still a lack of studies addressing the underlying mechanisms of these relationships. The flourishing literature has well established that emotional exhaustion constitutes the core component of burnout syndrome [16]. Therefore, a deep understanding of the relationships with negative emotional syndromes is paramount. In our first model, we assumed emotional exhaustion as a moderator of depression over time. As revealed by our findings, both T1 depression and emotional exhaustion had a significant effect on T2 depression. The findings of our moderation model also showed emotional exhaustion was a moderator of the longitudinal associations of depression among healthcare workers. Despite burnout and depression might share some underlying biological mechanisms such as specific profiles of inflammatory biomarkers [50], our findings study revealed that the former may influence the latter over time and, therefore, contribute to explaining why its prevalence varies widely across the studies, especially in time of pandemic [51]. Relatedly also, we found that emotional exhaustion acted as a moderator in repeated measures of anxiety. Although anxiety has received lesser attention than depression, it represents the most frequent symptom among healthcare workers [51]. Our findings lead to the consideration of emotional exhaustion as a factor that may increase depression and anxiety levels over time. Past research has taken into account several factors (for example, gender) as responsible for variety in burnout prevalence [52] but considering to a lesser extent depression and anxiety syndromes. This could be in part related to the perceived stigma affecting depression and other mental disorders [42].

As revealed by our results, emotional exhaustion may constitute a moderator for measures of anxiety over time, though more longitudinal research is still needed. Instead, relative to longitudinal measures of stress, our findings did not show a moderator role for emotional exhaustion. To explain this finding, we can argue that, historically, burnout has been considered as stemming from interpersonal stressors at work, and not vice versa [18]. Therefore, the moderator hypothesis we tested may be not appropriate on the grounds of these theoretical premises.

The findings of the current study have some relevant practical implications for future research and prevention of psychopathology among healthcare workers. First, this study confirmed the prolonged psychological impact of the pandemic. Negative emotional states as well as emotional exhaustion reported by healthcare workers during the early stage of the COVID-19 pandemic did not differ from what they experienced during the so-called second wave. Since this confirmed the view of the pandemic as a chronic stressor, healthcare authorities should maintain constant attention to the mental health of professionals. Taking into account staff burnout is necessary but not sufficient alone insofar as it tends to occur with depression, anxiety, and stress, even when the latter are measured at different times. A key component of burnout such as emotional exhaustion seems to moderate depression and anxiety over time. Hence, our findings could help to manage psychological interventions for healthcare workers assisting end-of-life cancer patients. Put another way, intervention to manage burnout may also have positive effects on concomitant psychiatric symptoms. While healthcare workers are more willing to accept the former, the psychological intervention may also work on the latter representing a difficult target since the associated stigma.

Future research should consider some limitations of this study. First, we adopted a convenience sample recruitment and, consequently, the results may not be entirely generalizable due to some variables not being well balanced and heterogeneity, such as profession type. Second, the use of self-report instruments for depression, anxiety, and stress did not allow for excluding pre-existing mental disorders with absolute certainty. Third, although other studies found similar prevalence, the attrition rate of 45% might indicate a selective dropout of some participants and, so, a potential bias.

## Conclusion

To sum up, our results indicate that emotional exhaustion behaves as a moderator of longitudinal measures of depression and anxiety among healthcare workers assisting patients with end-stage cancer. Taken together, these results point out the specificities in the relationship of emotional exhaustion with depression, anxiety, and stress. Researchers and specialists within the field of palliative care may find worthwhile our findings when assessing and preventing burnout syndrome among healthcare workers.

## Data Availability

The datasets used and/or analyzed during the current study available from the corresponding author on reasonable request.
